# Quantitative EEG and Verbal Fluency in DBS Patients: Comparison of Stimulator-On and -Off Conditions

**DOI:** 10.3389/fneur.2018.01152

**Published:** 2019-01-09

**Authors:** Florian Hatz, Antonia Meyer, Anne Roesch, Ethan Taub, Ute Gschwandtner, Peter Fuhr

**Affiliations:** ^1^Department of Neurology, Hospitals of University of Basel, Basel, Switzerland; ^2^Department of Neurosurgery, Hospitals of University of Basel, Basel, Switzerland

**Keywords:** Parkinson, DBS, quantitative EEG, automated artifact removal, verbal fluency

## Abstract

**Introduction:** Deep brain stimulation of the subthalamic nucleus (STN-DBS) ameliorates motor function in patients with Parkinson's disease and allows reducing dopaminergic therapy. Beside effects on motor function STN-DBS influences many non-motor symptoms, among which decline of verbal fluency test performance is most consistently reported. The surgical procedure itself is the likely cause of this decline, while the influence of the electrical stimulation is still controversial. STN-DBS also produces widespread changes of cortical activity as visualized by quantitative EEG. The present study aims to link an alteration in verbal fluency performance by electrical stimulation of the STN to alterations in quantitative EEG.

**Methods:** Sixteen patients with STN-DBS were included. All patients had a high density EEG recording (256 channels) while testing verbal fluency in the stimulator on/off situation. The phonemic, semantic, alternating phonemic and semantic fluency was tested (Regensburger Wortflüssigkeits-Test).

**Results:** On the group level, stimulation of STN did not alter verbal fluency performance. EEG frequency analysis showed an increase of relative alpha2 (10–13 Hz) and beta (13–30 Hz) power in the parieto-occipital region (*p* ≤ 0.01). On the individual level, changes of verbal fluency induced by stimulation of the STN were disparate and correlated inversely with delta power in the left temporal lobe (*p* < 0.05).

**Conclusion:** STN stimulation does not alter verbal fluency performance in a systematic way at group level. However, when in individual patients an alteration of verbal fluency performance is produced by electrical stimulation of the STN, it correlates inversely with left temporal delta power.

## Introduction

Deep brain stimulation of the subthalamic nucleus (STN-DBS) is widely used in advanced Parkinson's disease (PD) to treat motor complications. The subthalamic nucleus is the preferred target for DBS in most cases (1). STN-DBS improves motor manifestations in the limbs, while axial motor manifestations and language are improved variably, to a lesser extent, or not at all (2).

As for neuropsychological performance, verbal fluency (VF) performance is reportedly impaired by STN-DBS (3–7), while there is less evidence for GPi-DBS causing a decline in VF (8). The pathophysiological reason for this decline is still in debate (8). Chouiter et al. found that lesions by stroke or tumor of the left basal ganglia impair semantic and phonemic VF performance (9). However, to our current knowledge there is no study showing a direct long-term effect of precise microsurgical placement of electrodes on neuropsychological capacity. Interestingly, in a study by Isler et al. (10) reduction in cognitive flexibility after microsurgical penetration of the caudate nucleus recovered after 12 months. Anatomical studies in non-human primates have yielded evidence of a subdivision of the STN into a motor portion, an associative portion, and a smaller limbic portion (11, 12), while the associative portion is highly connected to the dorsolateral prefrontal and lateral orbitofrontal cortex. STN stimulation lessens the amount of language-related basal ganglia output via the thalamus and thus reduces thalamo-cortical drive (13). As striatal dysfunction is thought to induce set-shifting deficits by way of secondary dysfunction of the prefrontal cortex (4), this may partly account for decrease of VF after DBS. While it has been shown that the decline of VF after STN-DBS is an effect of the surgical procedure/perioperative activities (14), the influence of the electrical stimulation on VF performance is still controversial.

Both, the temporal and frontal lobes are involved in semantic and phonemic fluency tests. The left hemisphere is generally more important for VF than the right, and frontal lobes are more relevant for phonemic than for semantic fluency (15, 16).

Reduction of VF performance correlates with a reduction of median frequency or an increase of relative power in lower frequency bands in EEG (17).

In this study we aim to characterize the STN-stimulation-related changes in semantic, phonemic, and alternating fluency tasks and quantitative EEG (QEEG) measures (band powers, median frequency) in a group of PD patients. As VF is reduced by DBS, potentially by the stimulation itself, and as changes in VF performance are linked to frontal and temporal lobes, we expect a reduction of VF performance in the DBS-on compared to the DBS-off condition along with an increase of lower band power in frontal and temporal lobes.

## Methods

### Patients

Eighteen patients with STN-DBS were included. Sixteen completed the study protocol and were included in the analysis. Fifteen patients were right-handed and one was ambidextrous. Subjects characteristics are shown in Table 1. All of them underwent high-density resting-state EEG recordings (256 channels) and testing of VF in the DBS-on and DBS-off conditions. The phonemic, semantic, alternating phonemic, and alternating semantic fluency was evaluated RWT, Regensburger Wortflüssigkeits-Test, 2 min testing per task, no counting of errors, (18). Median age was 68.0 (IQR 60–71), 9 males and 7 females. Median duration of education was 14 years (IQR 12–16.5). Median years after first symptoms of PD were 12.5 (IQR 10.75–19). Patients were included 32 months (IQR 26–58) after STN-DBS operation and had a median levodopa equivalents dose of 562 (IQR 219–798).

**Table 1:** Subjects characteristics.

Age (years)	68.0 (59.2–71.8)
Education (years)	14 (12–16.5)
Years since diagnosis of PD (years)	12.5 (10.75–19)
Levodopa-equivalence-dose (mg)	562 (219–798)
Duration since DBS implantation (months)	32 (25.75–58.25)

*Median values and lower/upper quartiles are shown*.

### EEG Recording

After initial testing of VF performance, QEEGs from all patients were initially recorded with the DBS-on. For EEG recordings a 256-channel Geodesic DC-EEG System 300 was used. Sampling rate was set to 1,000 Hz, first high pass filter to 0.01 Hz. Impedances of EEG electrodes were kept below 40 kΩ. Subjects were seated comfortably in a reclining chair in a dimly lit, sound attenuated and electromagnetically shielded room. They were instructed to relax, but to stay awake and to minimize eye and body movements. After 12 min recording DBS was turned off and QEEG was recorded for additional 12 min, followed by VF testing. As for all subjects in the study STN-DBS consisted in a monopolar stimulation.

### EEG Post-processing

**Figure 1:**
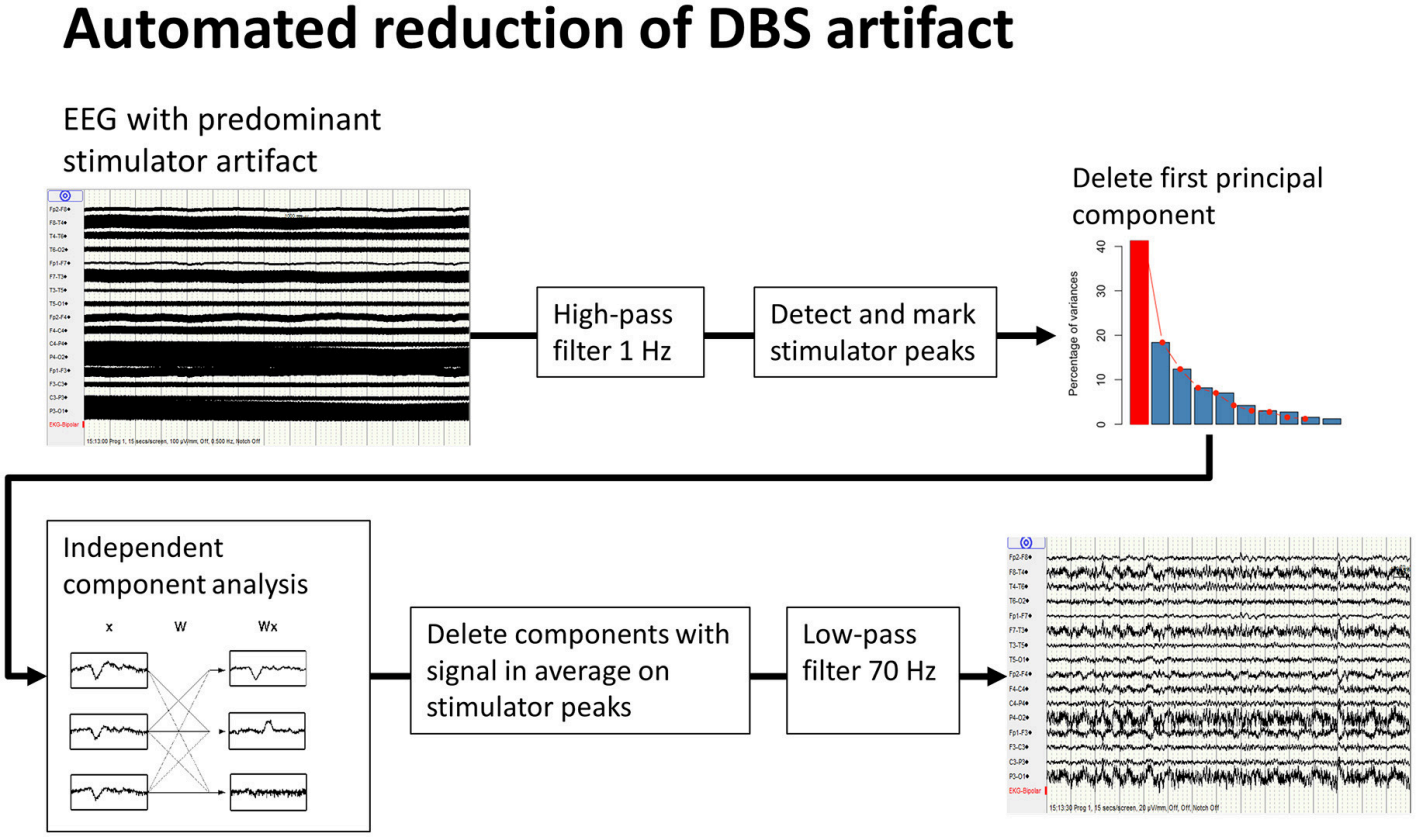
Workflow of automated reduction of DBS artifacts. A high-order least-square filter, high-pass at 1 Hz was applied first, followed by detection of the peaks of the stimulator artifacts. Second, a principal-component analysis was performed. A large part of the stimulator artifact is accounted for by the first principal component. This component was then deleted and the EEG reconstructed. Third, after an independent component analysis, all resulting components were averaged on the previously detected peaks for identification of the components, including DBS artifacts. These components were deleted and, again, the EEG was reconstructed. Finally, a high order least-square filter, low-pass at 70 Hz, was applied.

DBS-stimulation generates an artifact of considerably larger amplitude than the intrinsic brain signal recorded by EEG (19); the latter can only be analyzed once the former has been removed. Different methods of artifact removal have been proposed. The method described by Sun et al. for subtracting a reconstructed artifact is difficult to apply to real-life data (20). Lio et al. applied a combination of low-pass filter and a frequency-domain filter tracking outliers (21). Santillan-Guzman et al. proposed a temporal-frequency-domain filter (22). This method takes advantage of the known frequency characteristics of the artifact but does not exploit the similarity of signal shape at all of the recording electrodes due to the effect of volume conduction. We therefore used principal-component analysis to delete the first component, followed by an independent component analysis. These components were averaged using the DBS artifact as a trigger, and components with remaining signals after averaging were eliminated. Finally a 70 Hz low-pass filter (high-order, least-square filter) was applied (Figure 1). All steps for DBS-artifact elimination were integrated and performed in the toolbox “TAPEEG” (23), allowing fully automated artifact removal. Visually, frequency spectra for every patient in the ON- and OFF-condition were compared, showing a convincing reduction/elimination of the DBS artifact (Supplemental Figure 1).

### Inverse Solution (Frequency Domain)

Using a previously published method (24), resulting EEG data was re-referenced to average reference and bad channels were interpolated with spherical spline method. Power spectra were calculated from epochs of 4 s duration (spectral resolution 0.25 Hz) using Welch's method (20, 25). Source-space data was calculated by LORETA inverse solution for spectral data as described by Frei et al. (26), using a vector transposition matrix calculated with the software-package Cartool (27), based on the MNI brain atlas (28) and without using a normalization. The calculation was achieved using 5,011 solution points with subsequent reduction to 78 regions of interest (ROIs) based on the AAL atlas (29). According to previous studies by our group, median frequency and relative power in the delta- (1–4 Hz), theta- (4–8 Hz), alpha1- (8–10 Hz), alpha2- (10–13 Hz) and beta-(13–30 Hz) bands were calculated.

### Statistics

The relative band powers, median frequencies, and results of VF testing in the DBS-on and DBS-off conditions were compared with paired *t*-tests. The changes in relative power and VF tests were calculated (“value DBS-on” minus “value DBS-off”) and Spearman rank correlations calculated. Due to large intersubject power differences, relative power is the preferred measure for group data, while due to intrasubject stability of the EEG, absolute power is the preferred measure for longitudinal data within subjects. Permutation was used to correct for multiple testing and non-normal distributions of the resulting values.

The study was carried out in accordance with the recommendations of the Ethikkommission beider Basel (EKBB). The protocol was approved by the EKBB. All subjects gave written informed consent in accordance with the Declaration of Helsinki.

## Results

**Figure 2:**
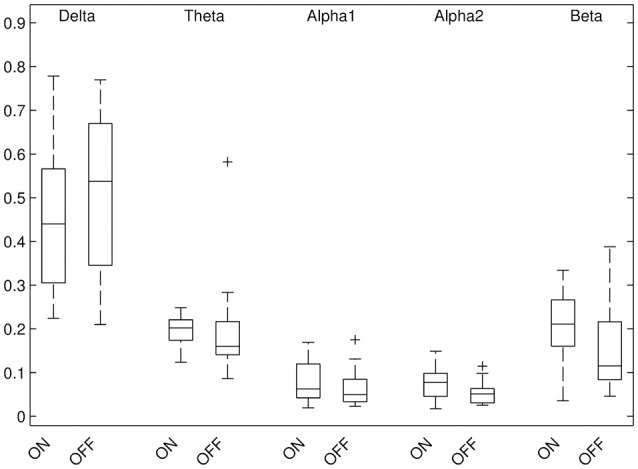
Results of global relative band power, comparison DBS-on vs. DBS-off.

**Figure 3:**
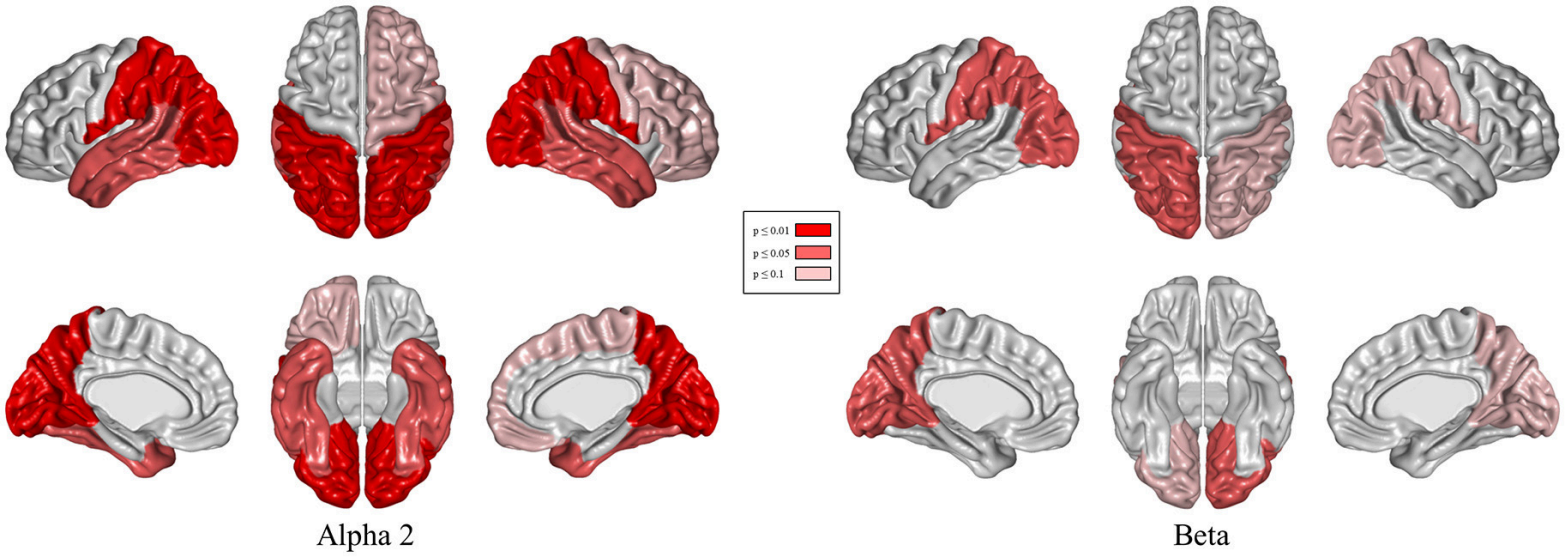
Topographical differences in relative alpha2 and beta power: DBS-on vs. DBS-off. Results of *t*-tests with permutation (red = regions with *p* < 0.01/light red = regions with *p* < 0.05/rose = regions with *p* < 0.1).

Results of relative band power are shown in Figure 2. In the DBS-on condition, the relative alpha2 power was higher in parieto-occipital regions bilaterally (Figure 3, *p* ≤ 0.01) and the relative beta power was higher in the left parieto-occipital region (Figure 3, *p* ≤ 0.05).

**Figure 4:**
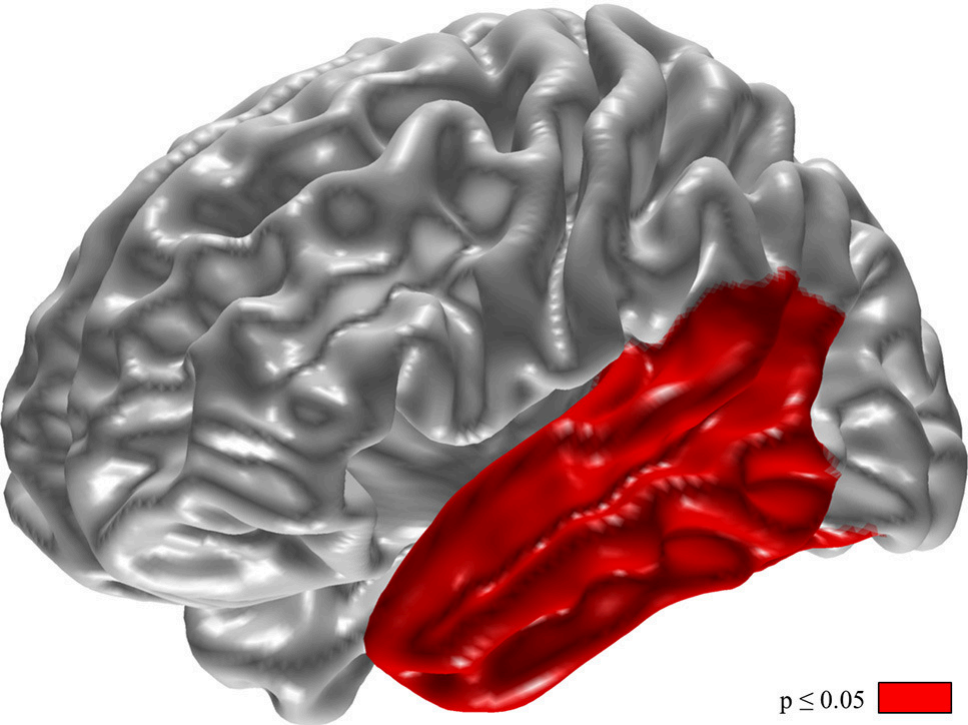
Inverse correlation of changes in phonemic verbal fluency and relative delta power. For calculation the difference of VF (VF with DBS-on minus VF with DBS-off) and relative delta power (delta with DBS-on minus delta with DBS-off). Results of *t*-tests with permutation (red = regions with *p* < 0.05).

Changes of relative delta power in the left temporal lobe and the phonemic VF were inversely correlated (Figure 4, *p* < 0.05).

**Figure 5:**
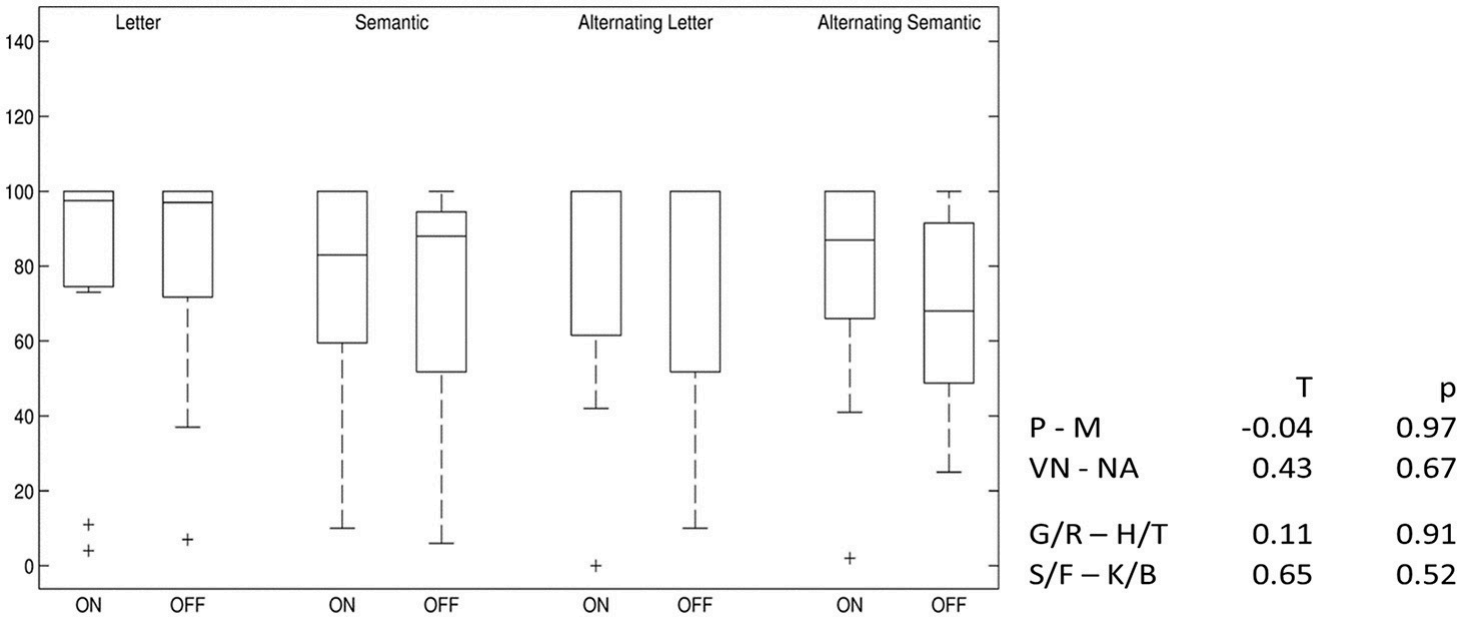
Differences in verbal fluency: DBS-on vs. DBS-off. Percent ranks of test results, boxplots showing median, upper and lower quartiles. P–M, comparison of phonemic fluency with “P” and “M” letters; VN–NA, comparison of semantic fluency with “Names” and “Food”; G/R–H/T, comparison of phonemic fluency with alternating “G” and “R” and alternating “H” and “T” letters; S/F–K/B, comparison of semantic fluency with alternating “Sport” and “Fruits” and alternating “Clothes” and “Flowers”.

Alternating semantic fluency performance slightly decreased after switching off stimulation, but this result is below statistical significance. For all other VF tests, no difference between the two conditions was found (Figure 5).

## Discussion

Switching off the STN-DBS did not improve VF performance in the present study in PD patients at least 6 months after operation. This finding accords with that of previous studies (14, 30) and supports the hypothesis that impairment in VF tests after operation is due to STN-DBS procedure rather than to the electrical stimulation. In addition to the surgical microtrauma, STN-DBS procedure includes anesthesia, changes in medication and sometimes post-operative delirium. Especially, the reduction of dopaminergic pharmacotherapy after the STN-DBS procedure may be an important factor, as VF performance is known to be ameliorated by dopaminergic drugs (31).

According to the present results, bilateral STN-DBS stimulation increases alpha2 and beta power in posterior regions in the resting-state EEG compared to the stimulator off condition. High alpha2 power is linked to increased capacity to initiate new tasks (32, 33) and probably facilitates switching between different tasks as tested in alternating VF. Alpha activity requires an intact thalamo-cortical loop (34), and its increase may reflect a partial functional normalization of this loop in the DBS-on condition, contributing to improved motor function and VF. This concept is compatible with the observation of a trend toward ameliorated alternating semantic fluency in the DBS-on state and is further supported by a previous study including 14 patients with PD, showing a slight positive effect of stimulation in the STN on phonemic fluency (30). However, this effect may be not strong enough to compensate for the decline of VF after STN-DBS procedure including a reduction of dopaminergic drugs.

The present result of increased beta power in the DBS-on state seems to contradict previous studies, which showed an improvement of motor function associated with a decrease of beta power over the sensorimotor, premotor and prefrontal cortex during STN stimulation and movement (35). However, in the present study the increase of beta power in the DBS-on situation occurs in resting state EEG and, therefore, the results are not directly comparable to studies analyzing event-related EEG alterations. Two MEG studies recorded also in resting state showed in contrast to the present results a decrease of beta band activity by STN-DBS (36, 37). While recordings in the study by Abbasi et al. took place 1 day after implantation and, therefore, were obtained in a different situation, the study by Luoma et al. with a latency of at least 3 months after implantation is better comparable to our results obtained at a latency of at least 6 months.

However, the present result is in line with the observation of a correlation between beta power in the paracentral region and motor function as well as sensorimotor integration after L-dopa intake (38) and with the results of a study by Cao et al. (39). The reason for these contradictory results is currently unclear; medication may play a role.

Changes of absolute delta power in the left temporal lobe and the phonemic fluency on the individual level correlate inversely. The fact that neither delta power nor phonemic fluency changes significantly between DBS-on and DBS-off conditions on the group level does not contradict such a correlation on the individual level as observed here. However, according to a post-stroke study using voxel-based volumetry phonemic fluency is a dysfunction of the left frontal rather than the left temporal lobe (15).

One limitation of this study is its small sample size. Speculations can be made about the underlying mechanisms of DBS-induced changes in brain rhythms, but no inferences can be drawn about therapeutic language effects in individual patients. For practical and ethical reasons, the time spent in the DBS-off condition was limited, and this necessitated the retesting of VF after only 12 min in off-time. Although this was longer than the 3 min off-time in the study by Yilmaz et al. (14), it may still not have been long enough for the effects of DBS on VF to disappear entirely.

## Data Availability

The EEG raw datasets of all patients in the study are not publicly available, as the signed informed consent by all patients does not include the possibility of the publication of these highly subject specific datasets. Raw results of the processed EEG datasets are available on demand. Requests to access the datasets should be directed to Peter Fuhr, peter.fuhr@usb.ch.

## Author Contributions

FH conceived and conducted the study, processed the EEG data, and drafted the manuscript. AM and AR conducted neuropsychological and linguistical testing of all patients. PF and UG initiated and designed the study, cared for the patients and critically reviewed the manuscript. ET had operated all patients and critically reviewed the manuscript. All authors read and approved the final manuscript.

### Conflict of Interest Statement

FH is supported by the Freiwillige Akademische Gesellschaft Basel. AM is supported by the Hedwig Widmer Foundation. UG's research is supported by Mach-Gaensslen-Foundation, Gossweiler Foundation, Parkinson Schweiz, Synapsis Foundation, Botnar Foundation. PF's research is supported by the Swiss National Science Foundation, Mach-Gaensslen-Foundation, Gossweiler Foundation, Parkinson Schweiz, Synapsis Foundation, Botnar Foundation, Freiwillige Akademische Gesellschaft Basel, Novartis Research Foundation, FreeNovation Foundation, as well as by unconditional research grants from Novartis, Roche, AbbVie, and Biogen. The remaining authors declare that the research was conducted in the absence of any commercial or financial relationships that could be construed as a potential conflict of interest.

## Supplementary Material

Supplemental Figure 1 Frequency spectra of single subject in DBS-off, DBS-on without artifact removal, and DBS-on after artifact removal (1 Hz highpass filter was applied before plotting).
