# Supplementary figures and images for: Quantitative EEG and Verbal Fluency in DBS Patients: Comparison of Stimulator-On and -Off Conditions
Source: Front Neurol. 2019 Jan 9;9:1152. doi: 10.3389/fneur.2018.01152 (PMC6333686; doi:10.3389/fneur.2018.01152)

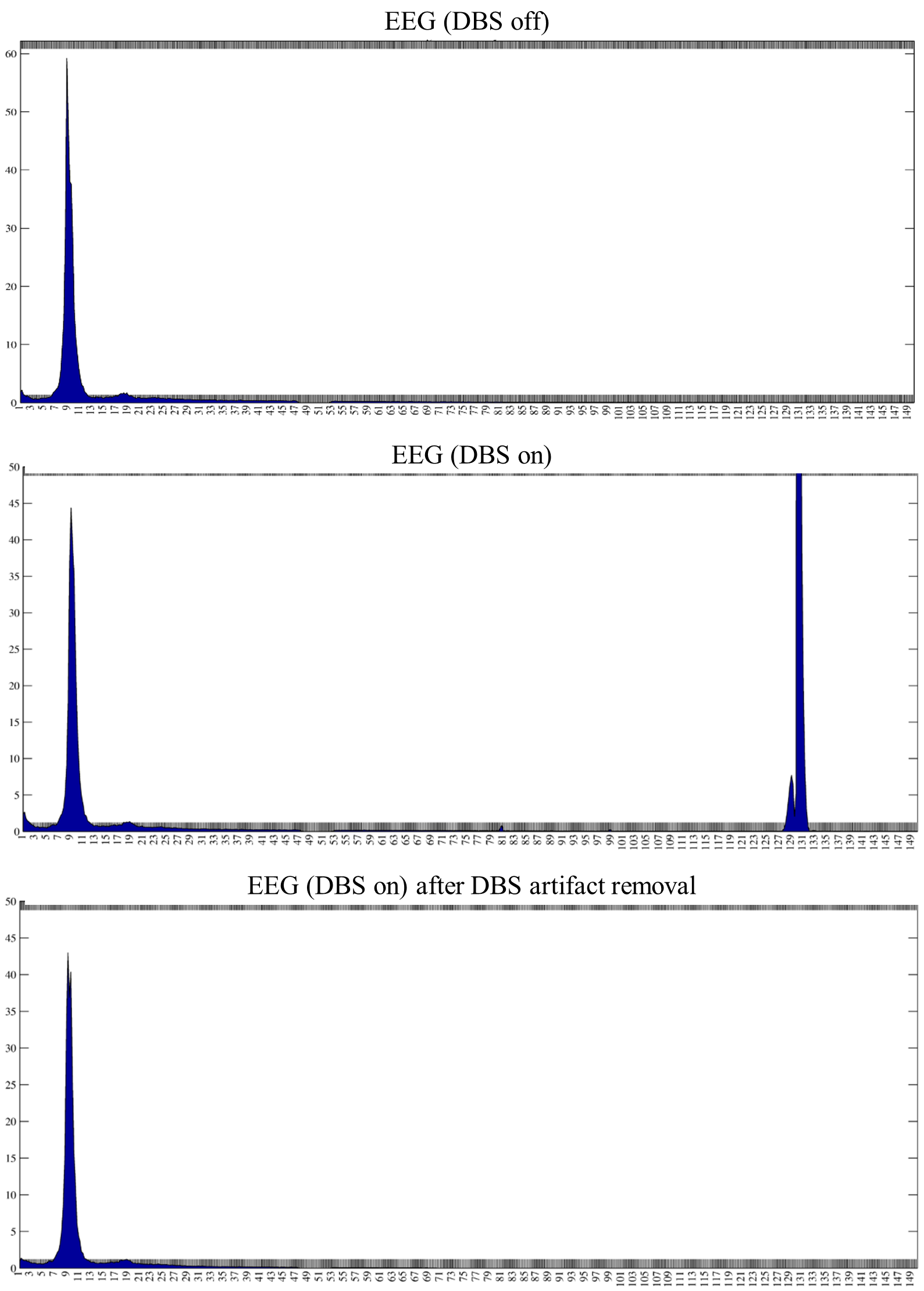

Supplement: Supplemental Figure 1 — Frequency spectra of single subject in DBS-off, DBS-on without artifact removal, and DBS-on after artifact removal (1 Hz highpass filter was applied before plotting). [file Image_1.TIF]
